# Disparities in breast cancer: a multi‐institutional comparative analysis focusing on American Hispanics

**DOI:** 10.1002/cam4.1509

**Published:** 2018-05-07

**Authors:** Zeina Nahleh, Salman Otoukesh, Hamid Reza Mirshahidi, Anthony Loc Nguyen, Gayathri Nagaraj, Gehan Botrus, Nabeel Badri, Nabih Diab, Andres Alvarado, Luis A. Sanchez, Alok K. Dwivedi

**Affiliations:** ^1^ Department of Hematology‐Oncology Maroone Cancer Center Cleveland Clinic Florida Weston Florida; ^2^ Division of Hematology‐Oncology Department of Internal Medicine Loma Linda University Health Loma Linda California; ^3^ Department of Internal Medicine Loma Linda University Loma Linda California; ^4^ Department of Internal Medicine Texas Tech University Health Sciences Center Paul L. Foster School of Medicine El Paso Texas; ^5^ Department of Biomedical Sciences Texas Tech University Health Sciences Center Paul L. Foster School of Medicine EL Paso Texas

**Keywords:** African American, Breast Cancer, Disparities, Ethnic differences, Hispanic, Hormone Receptor, Triple Negative

## Abstract

Breast cancer (BC) is the leading cause of cancer death in Hispanic/Latino women nationwide. Hispanic women are more likely to be presented with advanced disease and adverse prognosis subtypes. The aim of this study is to describe the clinico‐ pathological characteristics and disparities in breast cancer in this group at two tertiary care University‐based medical centers. After IRB approval, Cancer registry was used to analyze the variables of 3441 patients with breast cancer diagnosed and treated consecutively at two large tertiary University based medical and cancer center database centers in El Paso, TX and Loma Linda, CA between 2005 and 2015. Association between race/ethnicity and cancer type, stage, hormone receptor status and treatment option were investigated. Overall 45.5% of the patients were Hispanic (n: 1566) and those were more likely to be diagnosed at a younger age (57 years) similar to African Americans, more likely to have invasive ductal carcinoma type (82.7%) & triple negative disease (17.1%, 95%CI: 15% to 19%). 58.8% of Hispanics (95%CI: 56% to 61%) have hormone receptor (HR)+ & HER2− as opposed to 71% in non‐Hispanic White people. In addition, Hispanic individuals presented with advanced stages of BC (25.3%, 95% CI: 23% to 28%) similar to African American (25.4%), and had a lower proportion of lumpectomy (50%) similar to African American (50%). When compared to African American patients, Hispanic patients had a higher prevalence of triple negative BC (17.11% in Hispanics Versus 13.86% in African American). Conclusion: Hispanics had significantly higher relative risk of advanced stages at presentation (Relative Risk Ratio (RRR) = 2.05, *P* < 0.001), triple negative tumors (RRR = 2.64, *P* < 0.0001), HER2 + /HR ‐ disease (RRR = 1.77, *P* < 0.0001), and less HR+ /HER2− BC (RRR = 0.69, *P* < 0.0001). Hispanics and African Americans are diagnosed with breast cancer at a younger age, have a higher prevalence of Triple negative breast cancer, and are diagnosed at more advanced stages of disease. Increasing awareness and targeting minority populations for health promotion interventions, screening and early detection continue to be of paramount importance to reduce the burden of health disparities.

## Introduction

Breast cancer (BC) is the most commonly diagnosed cancer and the second leading cause of cancer death among women in the United States (US) 1, 2. In Hispanic American women, BC is the leading cause of cancer death 3. The Hispanic (Latino) population of the United States (US) constitutes 17% of the nation's total population making Hispanics the largest minority population 4 and the fastest growing population compared to other minorities in the United States 5.

Some studies have highlighted the impact of migration from low‐incidence countries such as Latin American countries to the United States (known as a high‐incidence country) on increasing the incidence rate of BC among immigrants 6, especially in the first generation of individuals who immigrated at a young age 7. Other studies have suggested significant disparities in the presentation of BC in Hispanic women in the United States compared to other groups related to more adverse prognosis, and increased prevalence of estrogen receptor and progesterone receptor (ER/PR)‐negative disease 8, 9, 10. A previous study by our group has suggested that BC in Hispanic women of Mexican origin is usually diagnosed at a younger age, with more advanced tumor stage, higher tumor grades, ER‐negative tumors, and a higher prevalence of breast cancer susceptibility gene 1 & 2 (BRCA1 and BRCA2) mutations than in non‐Hispanic White people 11. Another retrospective study in Hispanic women with BC in California showed that low socioeconomic status is associated with poor prognosis BC subtypes (triple negative, ER/PR negative) 12.

In this study, we aimed to better identify the clinical and pathological characteristics and possible disparities in BC in a large group of American Hispanics treated at two tertiary‐care University‐based medical centers in El Paso, Texas, and Loma Linda, CA, compared to other BC groups. El Paso, TX, is a large American‐Mexican border city with the majority of its residents being of Hispanic descent. Loma Linda University Medical Center serves a large Hispanic patient population. This multi‐institutional collaboration provides a desirable setting to study BC in Hispanics in two large states with Hispanics of primarily Mexican origin and allows the comparison of patients and tumor characteristics between Hispanic and non‐Hispanic White people as well other ethnic groups. The primary goal of this manuscript was to evaluate the differences in tumor characteristics between Hispanic and non‐Hispanic women. This study also explored the clinical and pathological characteristics between African Americans and other minorities as compared with non‐Hispanics and evaluated the similarities or differences with Hispanic patients.

## Methods

After obtaining IRB approval, we conducted a retrospective study and analyzed the characteristics of consecutive patients diagnosed with breast cancer and treated at two tertiary academic medical centers in Texas (Texas Tech University Health Sciences Center‐El Paso) and California (Loma Linda University Medical Center) between 1 January 2005 and 31 December 2015. All eligible patients from both sites were included in the study. No sampling strategies were adopted to generalize the results to El Paso, TX, and Loma Linda, CA, and thus, the weighting procedure was not used in the analysis. However, these two tertiary‐care centers are fairly representative of the target population as these two sites treat over a third of the whole breast cancer patient population in their respective areas. Patients’ ethnicity was self‐identified, and for simplicity, we categorized patients into the following four major ethnic categories: Hispanics (regardless of race), non‐Hispanic White people, African Americans, and other (including Native Americans, Asians). We used the tumor registries at both medical centers and the Texas Tech Cancer Clinical Research Core database in El Paso, TX, that includes clinical, pathological, and demographic characteristics of patients with breast cancer. Data were reviewed for accuracy by at least four co‐authors and verified by the corresponding and second authors.

We evaluated the following characteristics: mean age at diagnosis, gender, race, stage, histology of the tumor, ER, PR and HER2 status, cancer treatment, and type of surgery and compared these values between Hispanics and non‐Hispanics at both facilities and among Hispanics/Latinas in both facilities as well.

### Statistical analysis

Age was expressed using mean and standard deviation (SD) while other data were summarized using frequency and proportion. The distributions of clinical and tumor characteristics were presented for the entire cohort and according to ethnic groups (non‐Hispanic White people, Hispanic, African American, and other). The association between ethnicity and tumor characteristics was evaluated using multinomial logistic regression analysis after adjusting for age, diagnosis (infiltrating ductal carcinoma, lobular carcinoma, ductal and lobular carcinoma, or other), surgery (lumpectomy, mastectomy, and none), chemotherapy (yes, no), and radiotherapy (yes, no). Multinomial logistic regression was used for the outcome with multiple categories (more than two categories). The results of multinomial logistic regression analysis were presented using relative risk ratio (RRR) along with 95% confidence interval (CI) and *P*‐value. In addition, subgroup analysis was conducted using multinomial logistic regression analysis by comparing Hispanic patients in El Paso, TX, with those from Loma Linda, CA. This subgroup analysis was conducted to differentiate the tumor characteristics between Hispanics in El Paso, TX, versus Hispanics in Loma Linda, CA. All statistical analyses were carried out using SAS 9.4. P‐values less 5% were considered as significant results meaning the association was not observed due to chance alone.

## Results

A total of 3441 individuals with BC were included in this analysis. 1440 (41.8%) were non‐Hispanic White people, 1566 (45.5%) were Hispanics, and 202 (5.8%) were African Americans and 233 (6.7%) were other minorities. The distribution of patients’ characteristics for the entire cohort and according to different ethnic groups is summarized in Table 1. In the entire cohort, the majority of the patients were of age >50 years (70%), early‐stage cancer—stages I and II (80%), infiltrating ductal carcinoma (78.5%), ER+ (72.9%), PR+ (65.6%), and HER2− (77.6%). Non‐Hispanic White subjects were older (average age 60.7 years, 77% older than 50 years), were more represented in the California group (97%), and have a higher proportion of lobular or mixed ductal and lobular carcinoma types. Hispanics were more likely to be diagnosed at a younger age (average age 57 years, 36% younger than 50 years) similar to African Americans (average age 56 years, 33% younger than 50 years), and more likely to have invasive ductal carcinoma type (82.7%).

**Table 1:** Distribution of patients’ characteristics by ethnicity

Variables	Overall	Non‐Hispanic White	Hispanic	African American	Other	*P*‐value
	*N* (%)	*N* (%)	*N* (%)	*N* (%)	*N* (%)	
Baseline characteristics						
Diagnosis age—mean (SD)	58.15 (12.8)	60.73 (12.52)	55.87 (12.44)	56.67 (13.87)	59.06 (12.89)	<0.0001
Diagnosis age (years)						
≤50	1034 (29.77)	330 (22.92)	569 (36.33)	66 (32.67)	60 (25.75)	<0.0001
>50	2439 (70.23)	1110 (77.08)	997 (63.67)	136 (67.33)	173 (74.25)	
Gender						
Male	41 (1.18)	19 (1.32)	16 (1.02)	2 (0.99)	3 (1.29)	0.882
Female	3432 (98.82)	1421 (98.68)	1550 (98.98)	200 (99.01)	230 (98.71)	
Site						
El Paso, Texas	1266 (36.45)	40 (2.78)	1083 (69.16)	5 (2.48)	138 (59.23)	<0.0001
Loma Linda, California	2207 (63.55)	1400 (97.22)	483 (30.84)	197 (97.52)	95 (40.77)	
Diagnosis						
Invasive lobular	264 (7.63)	127 (8.82)	113 (7.27)	11 (5.45)	11 (4.72)	<0.0001
Invasive ductal and lobular	294 (8.49)	187 (12.99)	75 (4.82)	16 (7.92)	12 (5.15)	
Other	185 (5.34)	78 (5.42)	81 (5.21)	16 (7.92)	8 (3.43)	
Invasive ductal	2719 (78.54)	1048 (72.78)	1286 (82.7)	159 (78.71)	202 (86.7)	
Treatment profile						
Surgery type						
Lumpectomy	1801 (51.86)	789 (54.79)	782 (49.94)	101 (50)	116 (49.79)	0.0002
Mastectomy	1240 (35.7)	485 (33.68)	588 (37.55)	58 (28.71)	95 (40.77)	
Unknown/none	432 (12.44)	166 (11.53)	196 (12.52)	43 (21.29)	22 (9.44)	
Radiation						
No	1479 (44.03)	685 (47.7)	573 (39.35)	97 (48.02)	102 (43.78)	<0.0001
Yes	1880 (55.97)	751 (52.3)	883 (60.65)	105 (51.98)	131 (56.22)	
Chemotherapy						
No	1711 (50.94)	885 (61.63)	590 (40.52)	103 (50.99)	113 (48.5)	<0.0001
Yes	1648 (49.06)	551 (38.37)	866 (59.48)	99 (49.01)	120 (51.5)	
Tumor characteristics						
Cancer stage						
I	1210 (35.17)	601 (42.26)	462 (29.23)	64 (32.49)	70 (30.43)	<0.0001
II	1004 (29.19)	379 (26.65)	500 (32.07)	49 (24.87)	67 (29.13)	
III	448 (13.02)	112 (7.88)	264 (16.93)	31 (15.74)	39 (16.96)	
IV	242 (7.02)	76 (5.34)	130 (8.34)	19 (9.64)	14 (6.09)	
0	536 (15.58)	254 (17.86)	203 (13.02)	34 (17.26)	40 (17.39)	
Cancer stage						
3,4	690 (20.06)	188 (13.22)	394 (25.27)	50 (25.38)	53 (23.04)	<0.0001
0–2	2750 (79.94)	1234 (86.78)	1165 (74.73)	147 (74.62)	177 (79.96)	
ER						
Positive	2530 (72.85)	1107 (76.88)	1104 (70.5)	137 (67.82)	158 (67.81)	0.0006
Negative	861 (24.79)	298 (20.69)	427 (27.27)	60 (29.7)	68 (29.18)	
Unknown	82 (2.36)	35 (2.43)	35 (2.23)	5 (2.48)	7 (3.00)	
PR						
Positive	2277 (65.56)	1029 (71.46)	959 (61.24)	133 (65.84)	137 (58.8)	<0.0001
Negative	1117 (32.16)	379 (26.32)	573 (36.59)	64 (31.68)	88 (37.77)	
Unknown	79 (2.27)	32 (2.22)	34 (2.17)	5 (2.48)	8 (3.43)	
HER2						
Positive	632 (18.2)	206 (14.31)	184 (11.75)	32 (15.84)	24 (10.3)	0.0002
Negative	2694 (77.57)	1195 (82.99)	1288 (82.25)	165 (81.68)	196 (84.12)	
Unknown	147 (4.23)	39 (2.71)	94 (6)	5 (2.48)	13 (5.58)	
ER+, and/or PR+, HER2/neu‐						
Yes	2237 (64.41)	1025 (71.72)	921 (58.81)	126 (62.38)	145 (62.23)	<0.0001
No	1087 (31.3)	379 (26.32)	550 (35.12)	71 (35.15)	75 (32.19)	
Unknown	149 (4.29)	25 (2.5)	95 (6.07)	5 (2.48)	13 (5.58)	
ER‐, PR‐, and HER2/neu‐						
Yes	457 (13.16)	122 (8.47)	268 (17.11)	28 (13.86)	35 (15.02)	<0.0001
No	2864 (82.46)	1279 (88.82)	1203 (76.82)	169 (83.66)	185 (79.4)	
Unknown	152 (4.38)	39 (2.71)	95 (6.07)	5 (2.48)	13 (5.58)	
ER‐ and PR‐ and HER2/neu+						
Yes	178 (5.13)	51 (3.54)	99 (6.32)	11 (5.45)	16 (6.87)	0.0001
No	3143 (90.5)	1350 (93.75)	1372 (87.61)	186 (92.08)	204 (87.55)	
Unknown	152 (4.38)	39 (2.71)	95 (6.07)	5 (2.48)	13 (5.58)	

### Ethnic differences in treatment

Table 2 shows the overall adjusted association between ethnicity and type of surgery. Non‐Hispanic White (NHW) patients were more likely to receive lumpectomy (54.8%) followed by Hispanics and African American patients (50%). In the unadjusted analysis, we found that Hispanics were less likely to have lumpectomy as opposed to mastectomy (RRR = 0.82, *P* = 0.01) as compared with NHW. However, there was no difference in proportion of lumpectomy between African Americans and NHW (RRR = 1.07, *P* = 0.70). In the adjusted analysis, Hispanics tended to have more lumpectomy (RRR = 0.86, *P* = 0.10) compared to NHW after adjusting for other significant factors. Similarly, other minority groups were less likely to have lumpectomy (RRR = 0.74, *P* = 0.07) as compared to NHW after adjusting for potential confounders. A higher proportion of Hispanic patients received chemotherapy (59%) followed by African American patients (49%), than non‐Hispanic White patients (38%). 52% of NHW patients received radiotherapy which was similar to African Americans, while 61% of Hispanics received radiotherapy (*P* = 0.002). In the adjusted analysis, Hispanics were more likely to have radiation therapy (RRR = 1.21, *P* = 0.03) and chemotherapy (1.72, *P* < 0.001) as compared to NHW after adjusting for other cofactors. However, there were no differences in radiation therapy (RRR = 1.0, *P* = 0.99) and chemotherapy (1.19, *P* = 0.31) between African American and NHW. Compared to NHW, other minority individuals were more likely to receive chemotherapy (RRR = 1.45, *P* = 0.024) after adjusting for other potential confounders. There was no difference in proportion of radiotherapy (RRR = 1.03, *P* = 0.88) between other minority and NHW.

**Table 2:** Adjusted effects of ethnicity on tumor characteristics^a^

Outcome Variables	Hispanic vs Non‐Hispanic White (ref)	African Americans vs Non‐Hispanic White (ref)	Hispanic vs African Americans (ref)
	RRR (95% CI), *P*‐value	RRR (95% CI), *P*‐value	RRR (95% CI), *P*‐value
Cancer stage			
I	0.90 (0.71, 1.14), 0.409	0.77 (0.49, 1.22), 0.267	1.14 (0.72, 1.82), 0.558
II	1.26 (0.97, 1.63), 0.082	0.89 (0.53, 1.49), 0.674	1.37 (0.82, 2.28), 0.229
III	1.86 (1.33, 2.6), 0.0003	1.66 (0.89, 3.10), 0.106	1.09 (0.59, 2.00), 0.765
IV	1.71 (1.15, 2.53), 0.007	1.15 (0.56, 2.35), 0.692	1.47 (0.72, 2.99), 0.284
0 (ref)			
Cancer stage			
III, IV	1.69 (1.36, 2.11), <0.0001	1.75 (1.17, 2.63), 0.006	0.97 (0.65, 1.44), 0.887
0–2 (ref)			
ER+, and/or PR+, HER2/neu‐			
Yes	0.76 (0.64, 0.9), 0.002	0.76 (0.55, 1.06), 0.107	1.01 (0.73, 1.39), 0.951
No (ref)			
Unknown			
ER+, and/or PR+, HER2/neu+			
Yes	0.69 (0.55, 0.87), 0.001	1.03 (0.68, 1.56), 0.862	0.65 (0.43, 0.99), 0.047
No (ref)			
Unknown			
ER‐, PR‐, and HER2/neu‐			
Yes	1.98 (1.55, 2.52), <0.0001	1.37 (0.87, 2.16), 0.172	1.40 (0.90‐0, 2.16), 0.131
No (ref)			
Unknown			
ER‐ and PR‐ and HER2/neu+			
Yes	1.61 (1.12, 2.32), 0.01	1.46 (0.74, 2.88), 0.273	1.11 (0.57, 2.14), 0.744
No (ref)			
Unknown			

RRR, relative risk ratio; CI, confidence interval

a Adjusted for age at diagnosis, diagnosis (infiltrating ductal carcinoma, lobular carcinoma, ductal and lobular carcinoma, or other), chemotherapy (yes, no), surgery (lumpectomy, mastectomy, none), and radiotherapy (yes, no). (ref: Unknown category in each variable included in the analysis but related coefficients are not presented.)

### Unadjusted ethnic differences in prognosis factors

Non‐Hispanic White patients were less likely to have advanced stage of BC at presentation (13.2%, 95% CI: 12–15%), more likely to have hormone receptor‐positive (HR +) and HER2‐negative tumors (HER2−) (71.7%, 95% CI: 56–61%), and less likely to have triple‐negative BC (8.5%, 95% CI: 7–10%) compared to the other ethnic groups. Compared to non‐Hispanic White people and other ethnic groups, Hispanics were more likely to have triple‐negative disease (17.1%, 95% CI: 15% to 19%). 58.8% of Hispanics (95% CI: 56–61%) have ER/PR+ and HER2− tumors compared to 71% in non‐Hispanic White people. In addition, as noted in Table 1, more Hispanic individuals presented with advanced stages of BC (25.3%, 95% CI: 23–28%) compared to Whites, but the Prevalence was similar to that seen in African Americans and other minorities (25.4%). Specifically, more Hispanics, African Americans, and other minorities presented with stages III or IV BC at diagnosis (27%, 25.38%, and 23.04%), respectively, compared to non‐Hispanic White people (13%). However, Hispanics and African Americans tend to have a higher prevalence of stage IV BC on presentation (8.34% and 9.64%, respectively) compared to non‐Hispanic White people (5.34%) as well as other ethnic minorities (6.09%).

When compared to African American patients, Hispanic patients with BC were noted to have a higher prevalence of triple‐negative BC (17.11% in Hispanics vs. 13.86% in African Americans) and both groups have a higher prevalence of triple‐negative BC than non‐Hispanic White people (8.47%). However, African American individuals with BC have the highest prevalence of HER2 positive (HER2 + ) disease (15.84%) compared to all other groups.

### Adjusted ethnic differences in prognosis factors

Table 2 shows the differences among ethnic groups for cancer stage, and other tumor characteristics after adjusting for age, diagnostic type, and treatment profile. Compared to non‐Hispanic White people, Hispanics had significantly higher relative risk of advanced stages at presentation (RRR = 1.69, *P* < 0.001), triple‐negative tumors (RRR = 1.98, *P* < 0.0001), HER2 + /ER‐/PR‐ disease (RRR = 1.61, *P* = 0.01), and less ER+/PR+/HER2− BC (RRR = 0.76, *P* = 0.002). Hispanics had a higher relative risk of stages III and IV disease at presentation, as well as more triple‐negative breast cancer compared to any other ethnic group. Compared to non‐Hispanic White people, African Americans had higher relative risk of stage III BC (RRR = 1.66, *P* = 0.106) at presentation but to a lesser extent compared to Hispanics (RRR = 1.86, *P* = 0003). African Americans were more likely to have stages III and IV disease compared to non‐Hispanic White people (RRR = 1.75, *P* = 0.006). Although not statistically significant, African Americans had 24% less relative risk of having ER+/PR+ disease compared to non‐Hispanics White people (RRR = 0.76, *P* = 0.107) and more triple‐negative cases (RRR = 1.37, *P* = 0.172) albeit less when compared to Hispanics. There were no differences in advanced‐stage presentation between Hispanics and African Americans. Although not statistically significant, the relative risk of triple‐negative was found to be higher in Hispanics compared to in African Americans (RRR = 1.40, *P* = 0.131).

In the subgroup analysis among Hispanics, patients with breast cancer in El Paso, TX, were more likely to have advanced stages of BC (RRR = 2.15, *P* < 0.0001), less likely to have ER+/PR+ (RRR = 0.56, *P* < 0.0001), and more likely to have triple‐negative BC (RRR = 2.38, *P* < 0.0001) compared to the Loma Linda, CA, group.

## Discussion

This study confirms the significant ethnic disparities that exist between Hispanics and non‐Hispanics in BC, including more advanced stages at presentation, receiving less breast‐conserving surgery versus mastectomy, as well as possibly biological differences with increased prevalence of triple‐negative disease and younger age at presentation in Hispanics compared to non‐Hispanic White people, but with some similarities to African Americans despite some noted differences 9, 13, 14, 15. This is one of the largest studies to date that clearly identifies the biological and clinical disparities in BC characteristics in a large population of American Hispanics.

Most notably, Hispanics with BC appear to have the highest prevalence of stage IV disease at presentation with > 8% presenting with distant metastasis compared to around 5% in non‐Hispanic White people in both this study and nationwide data 16. They also appear to have the highest prevalence of triple‐negative breast cancer (17%) compared to all other ethnic groups. They also had a high prevalence of ER‐ and PR‐negative/HER2‐positive breast cancer (5.4%) compared to non‐Hispanic White people 2.

The strength of the study is the multi‐institutional collaboration between two large tertiary‐care centers in two states which gives it more representative weight for Hispanics overall. We noted that Hispanics in El Paso, TX, tend to have more advanced stages at diagnosis, suggesting possibly the effects of the lower socioeconomic status of this group in this federally designated underserved area of Texas; however, no other clinically significant differences were found between the two Hispanic groups.

Ethnic disparities related to breast cancer in Hispanics might be related to several factors noted previously in the literature including inferior participation of Hispanic women in cancer screening programs 17, prevalence of obesity and comorbidities, inadequate insurance coverage as well as less access, adherence, and possibly response to treatment 18, 19, 20, 21. Furthermore, prior studies have demonstrated that individuals without health insurance are less likely to have a usual source of health care and less likely to receive preventive services including cancer screening and are more likely to be diagnosed with late stages of cancer 22, 23. A study using data from the National Cancer Database examined the potential impact of health insurance status and stage at diagnosis for eight common cancers including breast cancer 22 among 843,177 patients sponsored by the American Cancer Society and the American College of Surgeons. For each cancer site, uninsured and Medicaid‐insured patients had the highest proportion of American Joint Committee on Cancer (AJCC) stages III and IV cancers at diagnosis, while those with private insurance and Medicare plus supplemental insurance had the lowest. While our study did not capture socioeconomic status (SES) specifically, over 50% of patients of patients from El Paso have Medicaid or are underinsured and that have likely contributed to advanced stages at presentation. A currently ongoing study is assessing, prospectively, adherence to cancer treatment among Hispanic women with BC.

In this study, we did not find any differences in locoregional treatment among any ethnic groups as compared to NHW. However, we noted disparities in systematic treatment with Hispanics receiving more chemotherapies and radiotherapies as compared to NHW even after adjusting for cancer stage and other covariates. While this finding could be related to more locally advanced stages at presentation necessitating chemotherapy and radiation therapy, other studies have noted that race/ethnicity, along with insurance and socioeconomic status (SES), does influence breast cancer treatment choices and possibly leads to inferior treatment in minority patients including Hispanics with early‐stage BC following diagnosis 9, 13. Friedman et al., 24 used multivariate logistic regression to assess the probability of definitive locoregional therapy, hormone receptor testing, and adjuvant systemic therapy among 662,117 White, Black, and Hispanic women diagnosed with invasive BC during 1998–2005 at National Cancer Database hospitals. After adjustment, African American (vs. White) women had less definitive locoregional therapy (odds ratio [OR], 0.91; 95% confidence interval [CI], 0.88–0.94), hormonal therapy (OR, 0.90; 95% CI, 0.87–0.93), and chemotherapy (OR, 0.87; 95% CI, 0.84–0.91). Hispanic (vs. White) women were also less likely to receive hormonal therapy while hormone receptor testing did not differ by race/ethnicity. Racial disparities persisted despite adjusting for insurance and SES.

Although this study included other ethnic groups (African Americans and other minority) in the analysis to explore the similarities and differences in tumor characteristics with Hispanics compared to non‐Hispanics, the primary focus of the study was to compare tumor characteristics between Hispanics and non‐Hispanics as these ethnic groups comprised a large proportion of our study sample.

One of the limitations of this study is that it did not capture the differences between US‐born and foreign‐born patients. The risk for developing breast cancer is higher in US‐born Hispanics than those born outside the United States 5, 25. It would be desirable in future studies to study the immigrant characteristics of breast cancer between these two groups. Also, the data did not capture survival data by stage, which would have been also valuable. In addition, we could not collect data on several confounding variables such as education, SES, insurance status, acculturation, body mass index due to retrospective nature of the study and capturing available data in the medical records. These covariates may affect the conclusion of the study and should be considered and collected in future studies.

Unfortunately, there is not enough information regarding the country of origin in our patent population group. However, as per 2010 US Census Bureau, over 80% of the total Hispanics comprises Mexican Hispanics from the states of California, Texas, and Arizona 4. This reflects that most of the Hispanics (over 80%) from both cities typically come from Mexican origin. Further, we do not anticipate any demographical or behavioral differences between Hispanics from two cites. However, there might be some unknown hospital practice differences between two sites. Similar to our study, number of publications from population‐based studies using SEER (Lu and Li, Siegel RL), CORI (Lieberman) assessed disparities in Hispanics compared to NHW and classified Hispanics irrespective of their country of origin or residing states 26, 27, 28.

Despite these limitations, this study has several noteworthy strengths: (1) It is a unique multi‐institutional effort addressing ethnic differences in breast cancer characteristics specifically focusing on Mexican American Hispanics as opposed to other studies that included a mixture of Hispanic groups or ethnic groups. (2) This study was conducted in two tertiary‐care centers and included a substantial sample size, which fairly represents the target population. (3) This study confirms that ethnic disparity exists between Hispanics and non‐Hispanics ethnic groups in terms of prognosis factors and treatment profile and shows that even after adjusting for treatment differences, age, and BC type, the ethnic differences persist for stages at presentation and biologic type, and therefore, (4) this study provides prevalence estimates of some important tumor characteristics in this minority population.

The purpose of the study was to determine whether breast cancer characteristics differ between Hispanic White people and non‐Hispanic White people and how these differences are inconsistent with other ethnic groups. However, the tumor characteristics might potentially vary by race or Hispanic subgroups due to many reasons including different genotypes, risk factors, and acculturation. To better understand Hispanics population and its heterogeneity, there is a need to explore race or subgroup differences in breast cancer characteristics among Hispanic population in future studies.

## Conclusion

Ethnic disparity exists between Hispanics and non‐Hispanics in regard to more advanced stages at diagnosis of BC, younger age at presentation and more prevalence of triple‐negative BC in Hispanics. Discrepancies were also noted in receiving less breast‐conserving surgery in Hispanics, which was similar to what is seen in other minorities including African Americans. Although there are multiple factors involved in these ethnic disparities, we believe that more targeted approaches such as culturally appropriate education to raise awareness of BC as well as improving access to screening and early diagnosis by improved healthcare coverage for minorities would be important first steps toward improving BC outcomes and reducing the overall burden of cancer in Hispanics and other minorities.

## Conflict of Interest

None declared.
